# Comparison of in silico predicted *Mycobacterium tuberculosis* spoligotypes and lineages from whole genome sequencing data

**DOI:** 10.1038/s41598-023-38384-3

**Published:** 2023-07-13

**Authors:** Gary Napier, David Couvin, Guislaine Refrégier, Christophe Guyeux, Conor J. Meehan, Christophe Sola, Susana Campino, Jody Phelan, Taane G. Clark

**Affiliations:** 1 Faculty of Infectious and Tropical Diseases, London School of Hygiene and Tropical Medicine, London, WC1E 7HT UK; 2 Institut Pasteur de la Guadeloupe, Les Abymes, Guadeloupe; 3 Université Paris-Saclay, Saint-Aubin, France; 4 CNRS, UMR ESE, AgroParisTech, 91405 Orsay, France; 5 DISC Computer Science Department, FEMTO-ST Institute, UMR 6174 CNRS, Univ. Bourgogne Franche-Comté (UBFC), 16 Route de Gray, 25000 Besançon, France; 6 Nottingham Trent University, Nottingham, NG1 4FQ UK; 7 IAME, UMR1137, Université Paris-Cité, INSERM, Paris, France; 8 Faculty of Epidemiology and Population Health, London School of Hygiene and Tropical Medicine, London, WC1E 7HT UK

**Keywords:** Bioinformatics, Genomic analysis

## Abstract

Bacterial strain-types in the *Mycobacterium tuberculosis* complex underlie tuberculosis disease, and have been associated with drug resistance, transmissibility, virulence, and host–pathogen interactions. Spoligotyping was developed as a molecular genotyping technique used to determine strain-types, though recent advances in whole genome sequencing (WGS) technology have led to their characterization using SNP-based sub-lineage nomenclature. Notwithstanding, spoligotyping remains an important tool and there is a need to study the congruence between spoligotyping-based and SNP-based sub-lineage assignation. To achieve this, an in silico spoligotype prediction method (“Spolpred2”) was developed and integrated into TB-Profiler. Lineage and spoligotype predictions were generated for > 28 k isolates and the overlap between strain-types was characterized. Major spoligotype families detected were Beijing (25.6%), T (18.6%), LAM (13.1%), CAS (9.4%), and EAI (8.3%), and these broadly followed known geographic distributions. Most spoligotypes were perfectly correlated with the main MTBC lineages (L1-L7, plus animal). Conversely, at lower levels of the sub-lineage system, the relationship breaks down, with only 65% of spoligotypes being perfectly associated with a sub-lineage at the second or subsequent levels of the hierarchy. Our work supports the use of spoligotyping (membrane or WGS-based) for low-resolution surveillance, and WGS or SNP-based systems for higher-resolution studies.

## Introduction

Tuberculosis is an infectious disease of high global burden caused by members of the *Mycobacterium tuberculosis* complex (MTBC), which includes nine human adapted lineages and four animal adapted lineages that are distributed phylo-geographically^1^. Although,  the MTBC is described as clonal, there is sufficient genetic variation to distinguish strain-types within members of the complex. Strain identification is crucial to addressing key epidemiological questions, from individual to global scales. Strain typing is informative in the investigation of transmission events and, in the wider context, provides valuable insight into the spread of MTBC variants, indicating potential differences between genotypes and phenotypes. For example, Beijing strains show lineage-specific associations with drug resistance^2^, and geographical ubiquity of lineages 2 (Beijing) and 4 (Euro-American) can be attributed to virulence and transmissibility^3^. Furthermore, strain typing at a higher phylogenetic resolution can reveal within-strain differences, such as between typical and atypical Beijing strains, which vary in geographical distribution, resistance, and virulence^4–6^. Advances in sequencing technologies, leading to whole genome sequencing (WGS) data, provide high-resolution strain typing, improved inference in transmission studies, enable the tracking of between- and within-lineage genotypic-phenotypic differences, and can assist with understanding drug resistance mechanisms.

Spoligotyping is a fingerprinting PCR technique^7^, which exploits the polymorphism harboured at the CRISPR locus of MTBC. It is based on the PCR amplification of 43 short unique sequences (termed spacers) contained between well-conserved 36-bp direct repeats. Since strains vary in the occurrence of spacers, each sample produces a distinctive spot pattern, which is then translated into a numerical code of 15 digits (octal code), leading to > 3,800 spoligotypes^8^. The spoligotyping nomenclature^8^ reflects the phylogeographical structure of MTBC, and its main families overlap with a SNP based barcoding system^9^, which was recently updated^1^. Both spoligotypes and SNP-based sub-lineages offer higher resolution than large deletion-based regions of difference (RD). However, the full extent of concordance between spoligotypes and sub-lineages needs to be established, potentially leading to improvements in both spoligotyping and sub-lineage barcoding of strain-types using WGS data. While spoligotyping has historically been used as an in vitro lab-based method, it is possible to generate spoligotype data from WGS data by looking at the presence of the 43 unique spacers. Of note is that the in vitro and in silico produced spoligotypes might not be 100% concordant due to the presence of IS6110 sequences in the CRISPR locus^10^. This issue was recently tackled by the development of the CRISPR-builder TB tool^11^, which improves accuracy of the CRISPR locus reconstruction and allows nucleotide variation in the spacers, direct repeats, or duplication events to be unraveled. Previous work has predicted spoligotypes in silico, implemented within the widely applied SpolPred software^12^. With at least 20-fold more *Mtb* WGS available since the development of SpolPred, we seek to assess the consistency of spoligotypes with the sub-lineage system^1^, and determine their global distribution. This goal is achieved by developing new software to in silico genotype isolates, called "Spolpred2", which predicts spoligotypes from raw sequence reads generated by several technological platforms. We incorporate the updated barcodes for spoligotypes and imbed "Spolpred2" within the TB-Profiler tool^13^, widely used to profile MTBC sub-lineages, strain-types, and drug resistance from WGS for clinical and surveillance applications.

## Results

### Global distribution of spoligotypes families

The dataset consisted of 28,436 M*. tuberculosis* isolates with WGS, drug susceptibility test and geographical source data, with lineages inferred using the TB-Profiler software (Table 1). The spoligotypes were predicted using the new Spolpred2 software, developed as part of this work (see MATERIALS AND METHODS) (Table 1). Most isolates were from the main global lineages (L4 50.3%, L2 25.9%, L3 11.2%, L1 10.1%), and the major spoligotype families identified were Beijing (L2; 30 spoligotypes; 25.3%), T (L4; 304 spoligotypes; 18.4%), LAM (L4; 187 spoligotypes; 12.9%), Central Asian Strain (L3; CAS; 125 spoligotypes; 9.3%), EAI (L1; 207 spoligotypes; 8.2%), though many samples had a spoligotype with an undesignated family (n = 3,318, 11.7%) (naming families stopped after WGS-based lineage systems were developed). A total of 100 unique (sub-)lineages and 2,991 unique spoligotypes were present. Whilst the isolates represent a convenience sample, of those that had an assigned geographic source (n = 26,209; 92.2%) they covered all World Health Organization (WHO) Regions, including Europe (36 countries; 39.6%), Africa (30 countries; 21.9%), Western Pacific (8 countries; 14.1%) and the Americas (14 countries; 11.2%). However, there were a modest number of isolates with an unreported country source (n = 2,227, 7.9%).

**Table 1:** *Mycobacterium tuberculosis* dataset.

Characteristic		No. members*	N (/28,128)	%	N (/24,661)**	%
Lineage	1	15	2868	10.1	2145	8.7
	2	5	7357	25.9	7252	29.4
	3	7	3195	11.2	2642	10.7
	4	52	14,298	50.3	12,128	49.2
	5	9	230	0.8	143	0.6
	6	10	128	0.5	78	0.3
	7	1	52	0.2	44	0.2
	Bovis	1	308	1.1	229	0.9
Spoligotype	Beijing	30	7197	25.3	7167	29.1
	T	304	5223	18.4	4829	19.6
	LAM	187	3672	12.9	3434	13.9
	Unknown	1827	3318	11.7	1002	4.1
	CAS	125	2641	9.3	2487	10.1
	EAI	207	2328	8.2	2061	8.4
	X	59	992	3.5	935	3.8
	H	92	959	3.4	827	3.4
	S	30	748	2.6	709	2.9
	Ural	38	458	1.6	414	1.7
	Bovis	32	267	0.9	229	0.9
	AFRI	30	205	0.7	173	0.7
	Other	28	422	1.5	394	1.6
WHO region	Europe	36	10,375	36.5	8690	35.2
	Africa	30	6162	21.7	5582	22.6
	Western Pacific	8	3971	14	3590	14.6
	Americas	14	3298	11.6	2996	12.1
	Unknown	-	2227	7.8	1933	7.8
	Southeast Asia	7	1878	6.6	1606	6.5
	Eastern Mediterranean	11	525	1.8	264	1.1

* sub-lineage, spoligotype, or number of countries; **excludes isolates with spoligotypes with frequency < 5.

**Figure 1:**
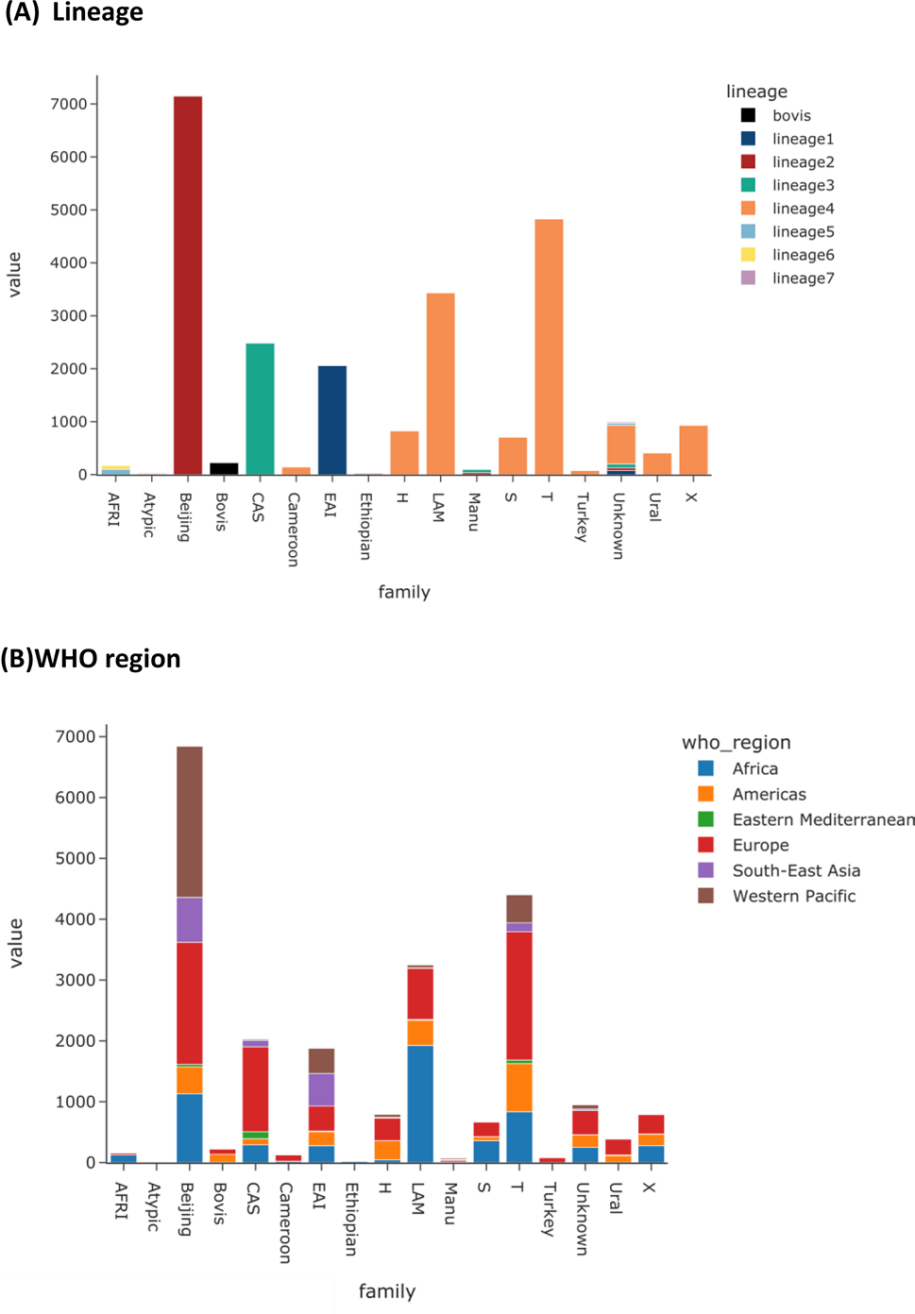
Spoligotype families and number of samples (n = 24,661); by (**A**) Lineage (L); (**B**) WHO region.

To improve the stringency of the analysis, all spoligotypes with < 5 isolates support were removed, resulting in 24,661 (86.7%) isolates, 96 (96.0%) unique lineages and 415 (13.9%) distinct spoligotypes (Table 1; Table 2; Fig. 1). This filtering task reveals the high number of rare spoligotypes (n = 3,775; see S1 Table for a list), with representation across most lineages (L4 57.5%; L1 19.2%; L3 14.6%; other 8.7%). After filtering (n = 24,661), the most frequent spoligotype families were Beijing (7,167; 29.1%), followed by T (4,829; 19.6%) and LAM (3,434; 13.9%), consistent with pre-filtering, but the proportion with unknown family decreased (n = 1,002; 4.1%) (Fig. 1; Table 1; S2 Table). The most common WHO geographical regions were Europe (n = 8,602; 38.2%), Africa (n = 5,579; 24.6%) and Western Pacific (n = 3,590; 15.8%) (Table 1; Fig. 1), also consistent with pre-filtered data. While many isolates occur in their expected geographical regions, such as Beijing strains in the Western Pacific and Southeast Asia, there is high variation in the reported source, reflecting the spread of *Mtb* since spoligotype labels were conceived, and the convenience nature of the sampling, which includes an emphasis on transmission studies or clinically relevant investigations.

**Table 2:** Spoligotypes with (sub-)lineages for *M. tuberculosis* (n = 24,661).

Lineage	Sub-lineage	N (%)	No. spoligotypes (%)*	No. families (%)
1	Overall	2145 (8.8)	70 (17.2)	13 (21.7)
	1.1	1214 (5.0)	48 (11.8)	10 (16.7)
	1.2	931 (3.8)	32 (7.9)	8 (13.3)
2	Overall	7252 (29.7)	22 (5.4)	4 (6.7)
	2.1	90 (0.4)	6 (1.5)	3 (5.0)
	2.2	7162 (29.3)	17 (4.2)	2 (3.3)
3	Overall	2642 (10.8)	62 (15.2)	8 (13.3)
	3	1765 (7.2)	54 (13.3)	6 (10.0)
	3.1	877 (3.6)	29 (7.1)	7 (11.7)
4	Overall	12,128 (49.6)	244 (60.0)	39 (65.0)
	4	109 (0.4)	13 (3.2)	3 (5.0)
	4.1	3348 (13.7)	96 (23.6)	21 (35.0)
	4.2	722 (3.0)	36 (8.8)	13 (21.7)
	4.3	3710 (15.2)	80 (19.7)	18 (30.0)
	4.4	1105 (4.5)	36 (8.8)	10 (16.7)
	4.5	385 (1.6)	38 (9.3)	13 (21.7)
	4.6	459 (1.9)	28 (6.9)	9 (15.0)
	4.7	251 (1.0)	17 (4.2)	9 (15.0)
	4.8	1771 (7.2)	60 (14.7)	16 (26.7)
	4.9	268 (1.1)	25 (6.1)	11 (18.3)
5	Overall	143 (0.6)	13 (3.2)	3 (5.0)
	5.1	118 (0.5)	10 (2.5)	3 (5.0)
	5.2	18 (0.1)	2 (0.5)	1 (1.7)
	5.3	7 (0.0)	1 (0.2)	1 (1.7)
6	Overall	78 (0.3)	4 (1.0)	2 (3.3)
	6	1 (0.0)	1 (0.2)	1 (1.7)
	6.1	13 (0.1)	2 (0.5)	1 (1.7)
	6.2	22 (0.1)	2 (0.5)	1 (1.7)
	6.3	42 (0.2)	3 (0.7)	2 (3.3)
7	Overall	44 (0.2)	3 (0.7)	2 (3.3)
Bovis	Overall	229 (0.9)	8 (1.9)	3 (4.8)

*Number of spoligotypes duplicated on some occasions due to presence in multiple lineages/sub-lineages.

### Spoligotype families and lineages

**Figure 2:**
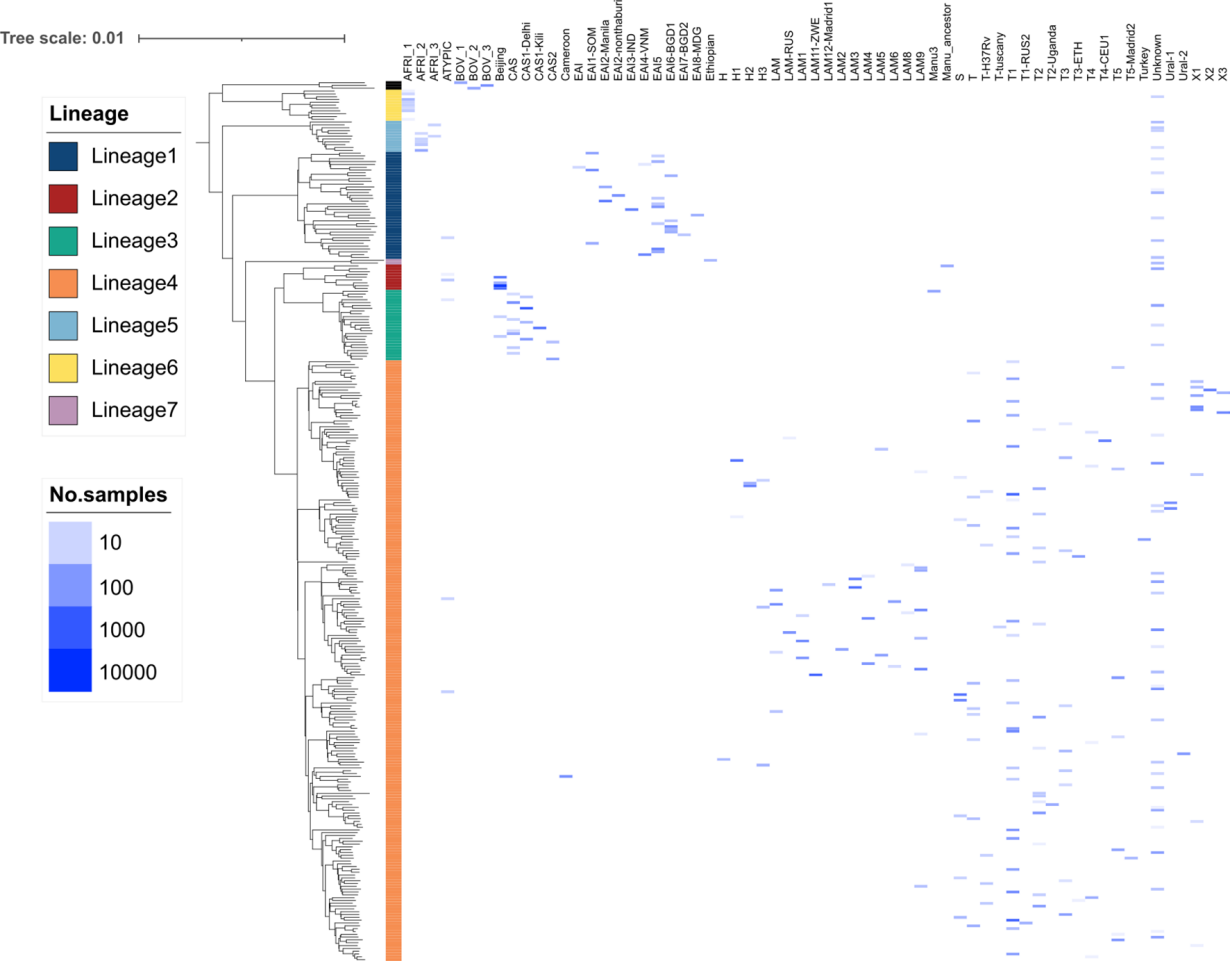
Spoligotype families and Lineages (n = 24,661). A representative phylogeny is used to position samples along the vertical axis. The horizontal axis represents families assigned using the spoligotype. The values on the heatmap represent the number of isolates with the exact sublineage/family combination.

There was a strong concordance between spoligotype family and main lineage among the 24,661 isolates (Table 2; S3 Table; Figure S1). At the main lineage level (L1 to L7), there were 408 (98.3%) spoligotypes appearing exclusively in their respective lineages. For example, the AFRI family only appears in isolates classed as L5 and L6. EAI, CAS, and Ethiopian families are exclusively found within L1, L3, and L7, respectively. Similarly, Cameroon, H, LAM, S, T, Turkey, and URAL spoligotype families appear only in L4, consistent with it being the most genetically diverse lineage (Fig. 2). There were however a few discrepancies, such as a very small proportion of isolates with a Beijing spoligotype family being classified as L1 (n = 1) or L3 (n = 19) (20/7167; < 0.4%) (S3 Table). These discrepancies could not be explained by low coverage in the direct repeat region. Isolates with the Manu spoligotype family were present in L2 (n = 38; 39.2%; Manu ancestor) and L3 (n = 59; 60.8%; Manu3). Most spoligotypes were found to be exclusive (sub-)lineages, and in many cases they made up only a relatively small proportion of that lineage's total samples (S3 Table). For example, spoligotype EAI2-Nonthaburi is only found in L1 but appears in only 5.8% of that lineage's total samples, and is known to be localized to Thailand^14^. EAI2-Nonthaburi is similar to the EAI-Manila spoligotype, originally found in the Philippines, and a dominant strain-type in that country ^15^. Conversely, as shown above, there are spoligotypes like Beijing which are highly prevalent in L2 (98.8%), but appear in two other lineages (S2 Table).

Subsequent analysis looked at spoligotypes within secondary (e.g., L4.2), tertiary (e.g., L4.2.2), quaternary (e.g., L4.2.2.1), and subsequent levels of lineages. At finer-scale levels of sub-lineages, there were decreasing numbers in perfect concordance with spoligotypes (second level: n = 300, 72.3%; third level: n = 288, 69.4%; fourth level: n = 271, 65.3%) (Figure S2). For comparison, the analysis was repeated using a set of proposed 68 spacers ^16^. Across the resulting 978 unique spoligotypes identified, the numbers assigned to single (sub-)lineages was greater than using 43 spacers (first level = 99.5%, second = 88.0%, third = 86.3%, fourth = 84.2%) (Figure S2). Finally, there were (43 spacer) spoligotypes with a high representation of isolates (n > 20) that offered high discrimination at fine-scale sub-lineage levels, including EAI2-Manila and EAI2-Nonthaburi (L1.2.1.2), Manu ancestor (L2.1), T4-CEU1 (L4.1.2), Turkey (L4.2.2.1), LAM1 and LAM2 (L4.3.4.1), and T2-Uganda (L4.6.1.1) (S4 Table). These spoligotypes could be used to update the lineage SNP barcode.

## Discussion

Our study aimed to characterize the global distribution of spoligotypes and correlate this with the lineage system developed previously^1^. To enable this work, a new rapid in silico spoligotyping software was developed with speed and flexibility in mind, and was integrated into the TB-Profiler analysis platform. The main improvements of Spolpred2 over the previous tool (SpolPred) include faster processing, with > 20- and > 500-fold speed improvements from fastq and BAM formatted files, respectively. Further, there is more flexibility with regards to input data types (e.g., gzipped fastq and BAM), the reporting of associated metadata (e.g., family names), and the ability to include custom spacer sets. The frequency of spoligotypes and their respective families confirm the known common spoligotype families, with representation from Beijing (SIT1), T (SIT53), LAM (SIT42), CAS (SIT26) and EAI (SIT236). The geographical distribution of spoligotype families followed known patterns, with T and LAM being most prevalent in Europe, Africa, and the Americas, AFRI in West Africa, and Beijing found across most geographic regions. Interestingly, there were 3,775 spoligotypes that were present in < 5 isolates, of which 1,647 (43.6%) had an assigned SIT in the SITVIT2 database, indicating that these are valid spoligotypes, although rare. In the future, it may be possible to assign family names to these unique and valid spoligotypes if sample sizes are greater. The remaining spoligotypes (56.4%) may represent novel forms or have been generated from isolates with spurious or low CRISPR locus coverage, although we employed quality control procedures on genome-wide coverage to minimize the latter.

Generally, there was a strong association of spoligotypes to lineage with the majority of spoligotype families associated exclusively to one of the major lineages. We did observe discrepancies in 20 isolates assigned as members of the Beijing spoligotype family, but with non-L2 lineages. These spoligotypes were manually verified, and could be the result of homoplasy events, which have been previously found using in vitro data^17^. In our data, some spoligotypes displayed a high degree of homoplasy, for example, the spoligotype designated as SIT4 had isolates belonging to 4.1, 4.2, 4.3, 4.4 or 4.8. As expected, the perfect concordance between spoligotype and lineage diminished as higher resolution sub-lineages were used for comparison, with only 65.3% of spoligotypes showing perfect concordance at the finest scale of sub-lineage assignment (4^th^ level). More often, spoligotypes may belong to more than one lineage, which indicates that spoligotypes may not be monophyletic, and could have arisen through convergent evolution, or that the sub-lineage comprises a higher resolution unit than the respective spoligotype(s). Conversely, there were some instances where a sub-lineage contained multiple major spoligotypes (e.g., EAI2-Manila and EAI2-Nonthaburi, both lineage 1.2.1.2), and hence the spoligotype represents the higher resolution unit for the related samples. In these cases, the sub-lineage system and corresponding SNP-barcode could be further refined to reflect this diversity. While current observations are based on large numbers of samples for the main lineages (L1-L4), fewer number of samples have been sequenced for the other lineages (L5-L9) and the relationships between WGS and spoligotype may not be as stable. These can be explored further through growing WGS datasets, and applications of phylogenetic analysis and in silico strain typing using the updated TB-Profiler tool.

## Conclusions

We have presented a method to predict in silico spoligotypes from WGS, called “Spolpred2”, which is fast and accurate. This software is freely available as part of the TB-Profiler package. Spoligotypes are useful in tracking the epidemiological spread of MTBC, but do not necessarily agree with the lineage system at more refined resolution of sub-lineages. We have clarified this relationship, which adds to the power of using a dual approach to strain typing.

## Material and methods

### Sequence data and processing

The input dataset consists of 28,436 isolates for which next generation sequences have been deposited on the ENA, and have been previously described elsewhere^13^. All sequence data was aligned to the H37Rv reference genome (NC_000962.3) using BWA mem software (v0.7.17). Variants were called using GATK HaplotypeCaller (v4.1.4.1 -ERC GVCF) and merged using the GATK CombineGVCFs tool. Variants were filtered to remove indels, SNPs in *pe/ppe* genes and those that had > 10% missing genotypes across isolates. Filtered variants were transformed to a multi-fasta format file, which was subsequently used as the input to phylogenetic reconstruction by iqtree software (v2.1.2 -m GTR + G + ASC). Lineage assignments were generated using TB-Profiler (v4.3.0). Alignment files in bam format were used for spoligotype generation using the algorithm described below.

### Spolpred2 algorithm

While the original algorithm relied on direct matching of hard-coded spoligotypes with input reads, the Spolpred2 spoligotype prediction tool is based on k-mer counting. The KMC3 tool ^18^ is used to count k-mers from either raw fastq, fasta, bam or cram format. A k-mer length equal to the length of the unique spacers (k = 25) is chosen. For bam and cram files, alignment against the H37Rv reference genome (AL123456.3)^19^ is assumed and only reads falling between positions 3,117,003 and 3,127,206 are analysed. A custom Python script then loads the counts and performs a direct look-up of the spacers, accounting for up to two mismatches. The presence or absence of a spacer is determined by comparing the counts against a minimum threshold. The threshold is selected to be 20% of the maximum spacer count. The presence/absence vector represents the binary spoligotype and is converted into an octal form. A limitation is that in silico and in vitro spoligotypes do not always match, due to potential interrupted spacers or direct repeat sequences leaving short sequences that are not detected by the algorithm. To obtain a full and precise reconstruction of the CRISPR locus, the semi-automatized application “CRISPR-Builder-TB” can be used^11^. Finally, the associated family and SIT are reported by performing a look-up in a CSV formatted file, which currently contains data for all isolates submitted to SITVIT2^20^. The code was integrated into TB-Profiler (v4.4.2)^13^ and can be invoked to perform spoligotyping only, or as part of the standard profiling pipeline, which also reports genotypic drug resistance and SNP-based (sub-)lineage profiles. Using a standard laptop with 8 Gb ram, Spolpred2 can profile from bam and fasta format files with 1000-fold coverage in < 10 s, whilst perform the same task on raw fastq files with up to 500-fold coverage in < 30 s. This translates to a 22- and 500-fold speed improvement from fastq and BAM files, respectively, when compared to the original SpolPred tool.

### Association of spoligotypes to lineages

Spoligotypes were inferred using Spolpred2 software across 28,436 MTBC samples with WGS data, location, and drug resistance phenotypes. Lineages and sub-lineages were inferred using the TB-Profiler tool, which implements a published barcode^1^. The number of (sub-)lineages within spoligotype families was estimated. As there were many spoligotypes in low numbers of samples and therefore offering little predictive power, those appearing in < 5 isolates were excluded. Since we were interested in the strength of association between spoligotypes and the various levels of the lineage system^1^, the lineages were parsed into a hierarchy for each round of analysis. For example, the first level analysed the association between each spoligotype and the main *Mtb* lineages 1–7. Next was the association between each spoligotype and the second level, represented by lineages 1.1, 1.2, 2.1, 2.2, and so on. If a sample did not have a classification at a certain level, the highest resolution classification was available used (e.g., L3 was regarded at a unit of classification at level 2, 3 and 4). A concordance correlation coefficient was used to test the statistical strength of association, where a score of 1 is assigned if a spoligotype is unique to a given (sub-)lineage, and anything less than 1 indicates that the spoligotype is found in at least one other isolate belonging to another (sub-)lineage.

### Ethical approval

No ethics approvals were required as all data is publicly available.

## Supplementary Information

Supplementary Information 1.

Supplementary Information 2.

## Data Availability

All data used in this work is publicly available. Spolpred2 software is available as part of TB-Profiler (https://github.com/jodyphelan/TBProfiler). Analysis scripts are available at https://github.com/GaryNapier/spolpred.
