# Supplementary material for: Comparison of in silico predicted Mycobacterium tuberculosis spoligotypes and lineages from whole genome sequencing data
Source: Sci Rep. 2023 Jul 13;13:11368. doi: 10.1038/s41598-023-38384-3 (PMC10345134; doi:10.1038/s41598-023-38384-3)

Supplementary figures

Figure S1

Phylogenetic trees for lineages showing the spoligotype spacer patterns and Lineages

(A) L1 and L7

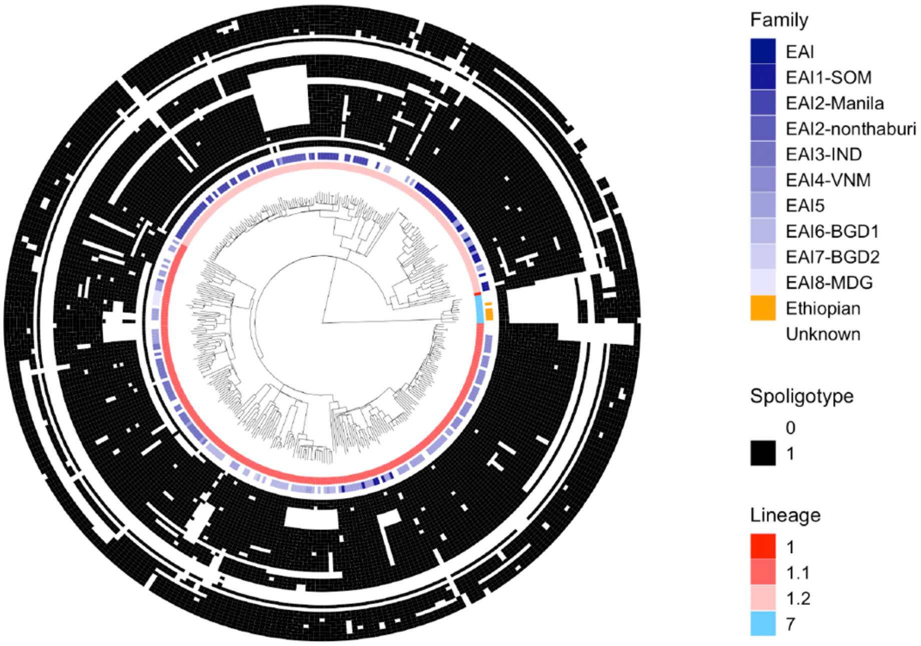

(B) L2

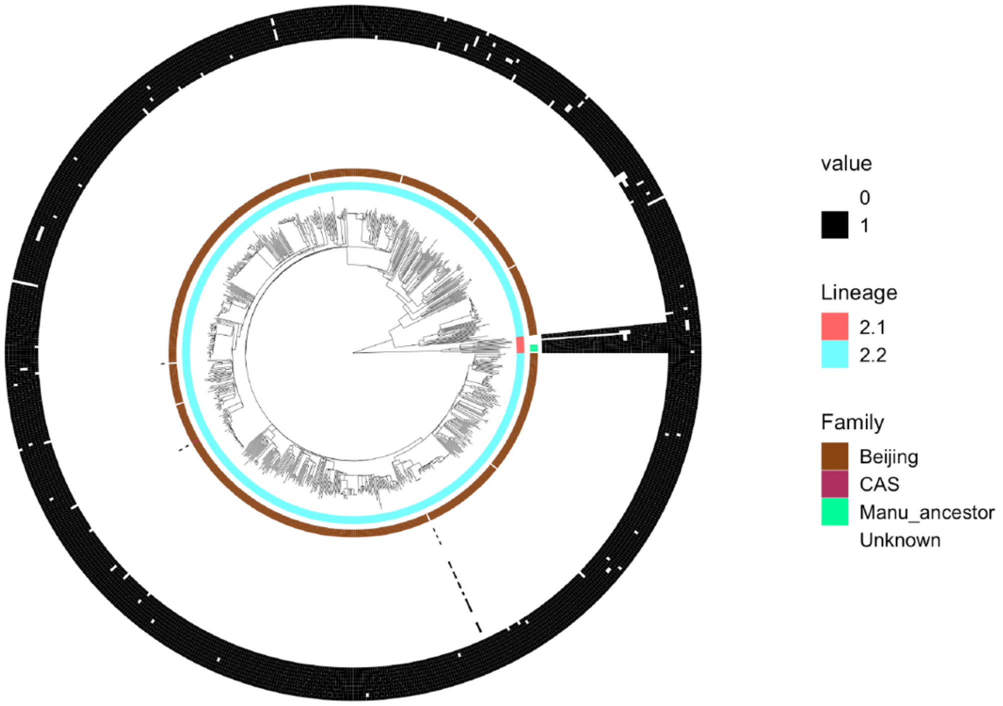

(C) L3

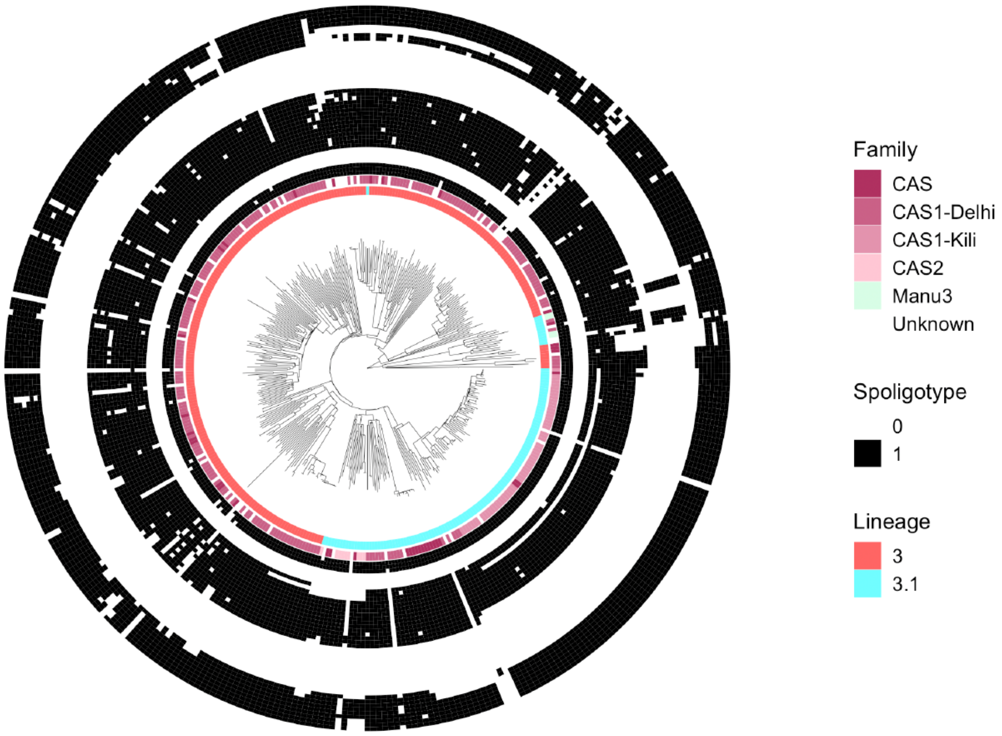

(D) L5, L6, L9, and La

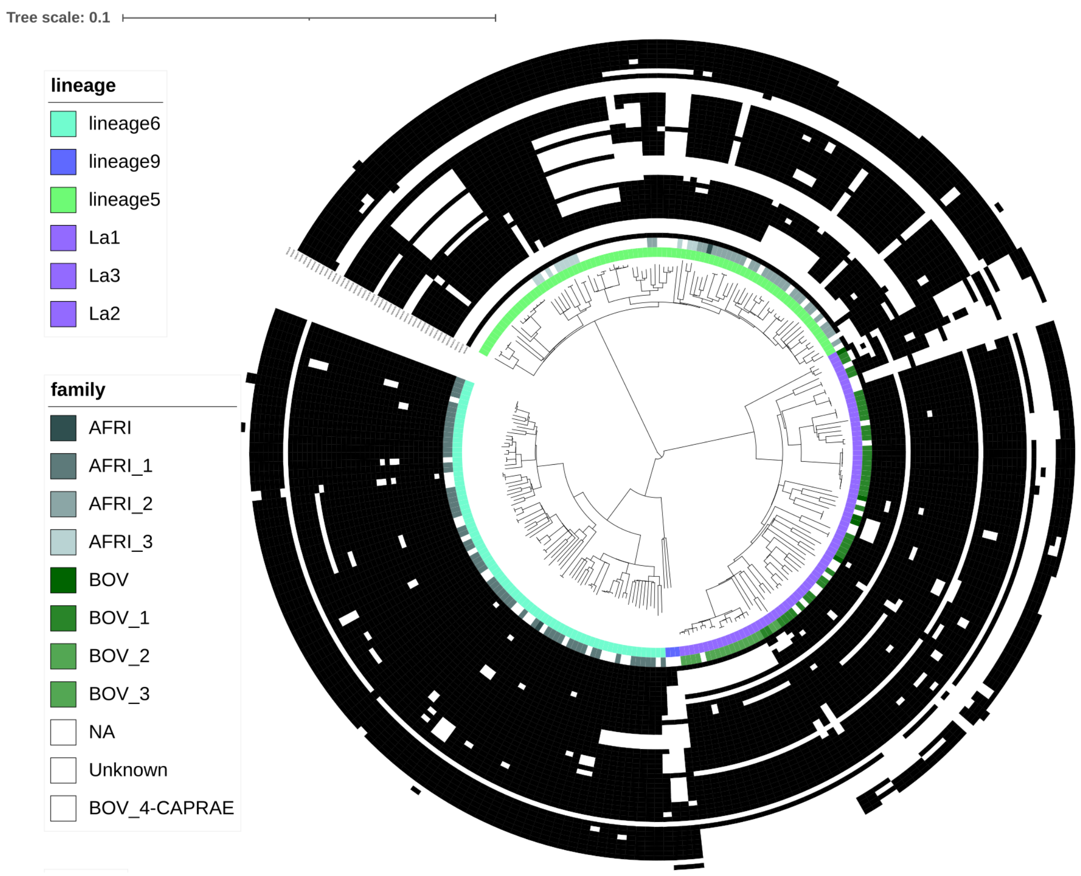

Figure S2

Frequencies of spoligotypes at each lineage level (n=24,661); (column 1) using the standard 43 spacer scheme and (column 2) the 68 spacer scheme

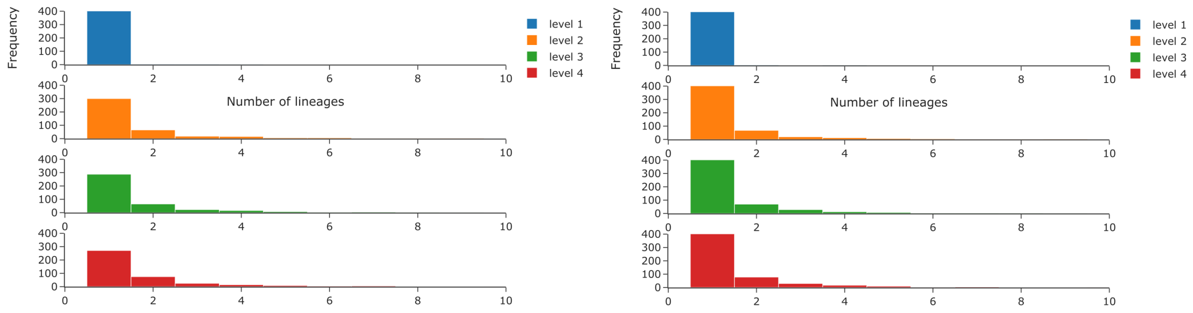

Supplement: Supplementary file 1 — Supplementary Information 1. [file 41598_2023_38384_MOESM1_ESM.pdf]
